# Miscellanea

**Published:** 1855-12

**Authors:** 


### MISCELLANEA.

In the University of Gottengen there are only 165 students of medicine, and in Heidelberg only 122.

*Died*, at Brighton, Nov. 19, 1855, aged 74, Thomas Copeland, Esq., author of the well-known work "On Some of the Principal Diseases of the Rectum."—At Brussels, M. Berger, Dean of the Accoucheurs of that city, who is said to have delivered 20,000 women.—At Paris, M. Louis Gondret, known by his advocacy of the use of ammoniacal preparations in amaurosis.—At Vienna, in the 64th year of his age, Dr. A. de Rosas, Professor of Opthalmology, and Director of the Opthalmic Clinic of the University. He spent thirty-six years in teaching his speciality.—At Petersburg, Va., J. F. Peebles, one of the editors of the Virginia Medical and Surgical Journal.—At Albany, on the 19th of Nov., in the 65th year of his age, Dr. T. R. Beck, of the Albany Medical College, author of "Beck's Medical Jurisprudence."—On the 8th of October, after a long and painful illness, the celebrated physiologist, François Magendie.—Prof. Johnson, of Durham, a well-known Chemist and writer on scientific subjects.

*A Hospital for Sick Children* has been opened in Philadelphia.

*The London Hospital for Consumption* has received since it was opened in 1846 3,656 in-patients, and 29,265 out-patients.

*Anæsthetics in the Austrian Army.*—The Army Medical Officers are ordered, in future, to employ for the purpose of inducing anæsthesia, a mixture consisting of one part chloroform and nine parts ether, this being the proportion long employnd by Dr. Weiger, a Vienna Dentist.

*A Consumption Hospital*, the first of the kind in the United States, has been recently organized in New York.

